# Unraveling the Interfacial Carrier Behavior in PtSe_2_–MoSe_2_ Heterostructures: Insights from Combined Pump‐Probe Spectroscopy and Scanning Tunneling Microscopy

**DOI:** 10.1002/advs.202500598

**Published:** 2025-03-07

**Authors:** Weili Zhang, Hu Chen, Jiangbo Peng, Xiaoguang Pan, Hangxin Bai, Fangli Jing, Hailong Qiu, Zhanggui Hu, Yicheng Wu, Hongjun Liu

**Affiliations:** ^1^ Tianjin Key Laboratory of Functional Crystal Materials Institute of Functional Crystals School of Materials Science and Engineering Tianjin University of Technology Tianjin 300384 China

**Keywords:** 2D heterostructure, charge transfer, pump‐probe spectroscopy, scanning tunneling microscopy/spectroscopy, transition metal dichalcogenides

## Abstract

Interfacial carrier behavior is vital for modifying the optoelectronic performance of 2D materials with atomical thickness, yet understanding exciton dynamics within hetero‐phase heterojunctions at the nanoscale remains elusive. Here, carrier dynamics at the interface in molecular beam epitaxy grown 1T PtSe_2_–1H MoSe_2_ heterostructures are revealed by ultrafast pump‐probe spectroscopy, and the corresponding mechanisms are studied by combining scanning tunneling microscopy/spectroscopy (STM/STS). The difference in exciton lifetimes and signal intensities in the heterostructures at the energies larger and narrower than the bandgap of MoSe_2_ demonstrates both electrons and holes transfer at the interface of PtSe_2_ and MoSe_2_ monolayers. Such bipolar transfer is interpreted through both the type‐I (lateral) and type‐II (vertical) band alignments revealed by STS measurements across the heterojunctions interface. Besides, it also shows that the band alignment modification of in‐plane heterojunctions by a specific conductive substrate can fairly alter the exciton dynamics at the interface. This work provides a comprehensive insight into the carrier dynamics in PtSe_2_–MoSe_2_ heterostructures with high spatial and temporal resolutions and may be beneficial for their design in optoelectronic devices.

## Graphical abstract

Combining ultrafast pump‐probe spectroscopy and scanning tunneling microscopy/spectroscopy, the excited carrier dynamics of molecular‐beam‐epitaxy‐grown PtSe_2_–MoSe_2_ heterostructures are revealed. These two types of heterointerfaces corresponding to different band alignments can give a well interpretation of the transient spectra under different excitation wavelengths, demonstrating the different charge transfer at the interface of the hetero‐phase heterojunction.

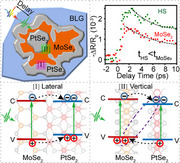

## 1 Introduction

The advent of 2D semiconductor heterostructures based on transition metal dichalcogenides (TMDs) with diverse band alignments has spawned numerous newfangled physical phenomena, paving a new pathway for designing target devices.^[^ ^1^ ^]^ Due to the atomical thickness, carriers at the interface of semiconductor heterojunctions can strongly modify the optical, electronic, and optoelectronic performance of the devices based on them. Hence, understanding photo‐excited carrier dynamics at the heterojunction interface is crucial for the design of heterostructures‐based devices. During the past years, the interfacial carrier transfer in these 2D semiconductor heterostructures has been extensively studied using pump‐probe spectroscopy.^[^ ^2^ ^]^ The complex excited‐state interactions have been more precisely elucidated through a three‐pulse sequence pump‐probe configuration.^[^ ^3^ ^]^ In‐depth research on excited‐state carriers in semiconductors has significantly extended their potential applications in optoelectronic devices.^[^ ^1a^ ^]^ Equally important for clarifying the mechanisms governing the performance of photo‐induced excitonic states would require access to the interfacial band alignment of the heterojunctions, which in most reports were mostly given by density functional theory (DFT) calculations.^[^ ^2a,c,d^ ^]^ However, DFT studies have revealed that variations in interlayer spacing not only modulate the bandgap,^[^ ^4^ ^]^ but also impact the electronic performance.^[^ ^5^ ^]^ Moreover, the Fermi level for TMDs can be readily shifted by the substrate, both strain and defects at the interface can also influence interfacial electronic structures.^[^ ^6^ ^]^ Hence, it is crucial to know the precise atomic structures and band alignment at the interface on a specific substrate. Scanning tunneling microscopy/spectroscopy (STM/STS) has been demonstrated to be a powerful tool to reveal interfacial electronic structures in heterojunctions at atomic scale.^[^ ^6^, ^7^ ^]^ Full studies with high spatial and temporal resolutions by combining pump‐probe spectroscopy with STM/STS are expected to provide an in‐depth comprehension of the excitonic dynamics and electronic performance at the interfaces of heterostructures.^[^ ^8^ ^]^

Most studies have been oriented toward the group‐VI layered TMDs, such as 1H‐MoSe_2_, renowned for its direct bandgap,^[^ ^9^ ^]^ extraordinary light absorption capabilities,^[^ ^10^ ^]^ and large exciton binding energy.^[^ ^11^ ^]^ However, the heterostructures based on them are limited in further application in optoelectronic devices, due to their poor performance in spectral response and carrier mobility.^[^ ^12^ ^]^ And most studied heterostructures based on group‐VI TMDs have the same H‐phase crystal structure. With the development of TMDs, 1T‐PtSe_2_ has shown many distinct advantages, such as widely tunable bandgap,^[^ ^13^ ^]^ anisotropic carrier mobility, and air‐stability,^[^ ^14^ ^]^ which expands the application of TMDs in mid‐infrared photodetection regime.^[^ ^15^ ^]^ These favorable characteristics would make the PtSe_2_‐based TMDs hetero‐phase heterostructure to be a kind of promising material with enhanced photophysical properties. Recently, plenty of PtSe_2_‐based heterostructures have been fabricated by chemical vapor transport,^[^ ^16^ ^]^ molecular beam epitaxy (MBE),^[^ ^17^ ^]^ mechanical exfoliation,^[^ ^18^ ^]^ etc. Among them, ultra‐high vacuum MBE is believed to be an excellent way to construct heterojunctions with ultra‐clean interfaces due to its impurity‐free growth conditions. Furthermore, these PtSe_2_‐based heterostructures have shown some attractive properties, e.g. interlayer charge transfer,^[^ ^16^, ^18^ ^]^ effective modulation of transport feature,^[^ ^16^, ^17^ ^]^ broad photoresponse,^[^ ^16^, ^19^ ^]^ and tunable Rashba effect.^[^ ^20^ ^]^ Although the device fabrication has made some progress with PtSe_2_‐based heterostructures,^[^ ^16^, ^17^, ^19^ ^]^ most studies on the intrinsic characteristics focused on DFT, X‐ray photoelectron spectroscopy (XPS), and Kelvin probe force microscopy, etc.^[^ ^16^, ^18^ ^]^ Some key problems, such as structure‐property relationships between excited carrier dynamics, atomic structures, and electronic properties at the interface, are still lacking for PtSe_2_‐based hetero‐phase heterostructures.

Herein, we fabricated monolayer (ML) 1T PtSe_2_–1H MoSe_2_ heterostructures grown by MBE, and the component evolution of the as‐grown sample was confirmed by reflected high‐energy electron diffraction (RHEED) pattern, Raman spectroscopy, and XPS. The carrier dynamics, as well as electronic structures, were studied with high temporal and spatial resolutions via both femtosecond time‐resolved pump‐probe spectroscopy and STM/STS. The transient reflectance (TR) spectroscopy and photoluminescence (PL) experiment revealed the existence of interfacial carrier transfer between PtSe_2_ and MoSe_2_, and the transfer mechanism can be interpreted with the interfacial band alignment by band profiles from STS measurements. Our work serves as a proof‐of‐concept study of PtSe_2_–MoSe_2_ heterostructures, which contributes to the fabrication of optoelectronic devices on this basis. Such high‐resolution measurements may be universally employed for the elaborate characterization of interfacial carrier dynamics of semiconductor heterostructures.

## 2 Results and Discussion

### 2.1 Growth and Optical Characterization of Heterostructure

**Figure 1:**
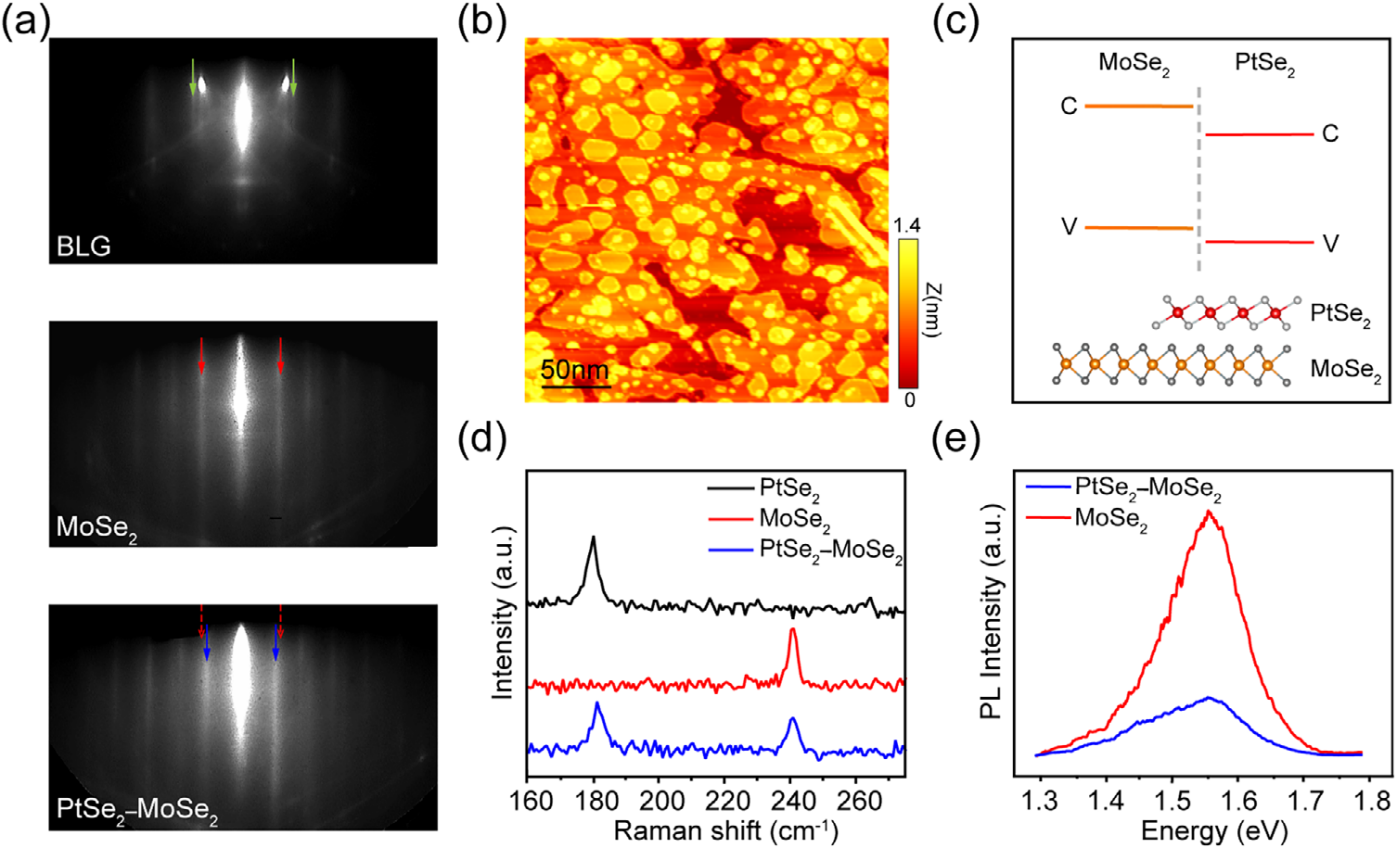
Growth of atomically thin PtSe_2_ **–**MoSe_2_ heterostructures by MBE. a) RHEED patterns of bilayer graphene on a 6H‐SiC (0001) substrate, MoSe_2_ film, and after growing PtSe_2_ on MoSe_2_, respectively. b) A STM topographic image of a PtSe_2_ **–**MoSe_2_ heterostructured sample. V_b_ = 3.5 V, I_t_ = 10 pA. c) The predicted alignment of the conduction (C) and valence (V) bands of the PtSe_2_ **–**MoSe_2_ heterostructure, which forms a type‐II heterojunction. The bottom inset is a schematic diagram of the corresponding heterostructure. d) Raman spectra of pure PtSe_2_ (black), pure MoSe_2_ (red), and heterostructure (blue). e) PL spectra of MoSe_2_ monolayer and PtSe_2_ **–**MoSe_2_ heterostructure grown on bilayer graphene. The results show the same center of peak position (1.55 eV), regardless of whether PtSe_2_ **–**MoSe_2_ heterostructure (blue) or pure MoSe_2_ (red).

ML PtSe_2_–MoSe_2_ heterostructures were grown on a bilayer graphene (BLG) substrate by MBE. During the growth, continuous lattice parameter changes were monitored by RHEED, as shown in **Figure** **1**a. Based on the measured intervals of the stripes, the lattice constants of MoSe_2_ and PtSe_2_ were calculated to be 3.28 and 3.73 Å, consistent with reported values.^[^ ^21^ ^]^ For more details about the growth refer to the Section S1 (Supporting Information). A large‐scale STM image shows that both lateral and vertical PtSe_2_–MoSe_2_ heterostructures can be observed on the BLG, and the majority of the as‐grown samples were vertically stacked in Figure 1b. Figure 1c illustrates the predicted band alignment of the heterostructure,^[^ ^16^, ^20^ ^]^ where the conduction band minimum (CBM) and the valence band maximum (VBM) lie in PtSe_2_ and MoSe_2_ layers, respectively. The bottom panel depicts the crystalline structure of hetero‐phase (1T/1H) heterostructure. Raman spectra on the as grown sample show both E_g_ mode of 180 and A_1g_ mode of 240 cm^−1^ in Figure 1d, corresponding to those for pristine PtSe_2_ and MoSe_2_, respectively.^[^ ^13^, ^22^ ^]^ Furthermore, the composition of the PtSe_2_–MoSe_2_ heterostructure was examined by XPS testing, and the core energy levels of Se, Mo, and Pt atoms in the heterostructure were essentially identical to those of the pure MoSe_2_ and PtSe_2_ samples (Figure S1, Supporting Information). These further confirm the successful growth of the heterostructure. To gain more information about the electronic structures of the heterostructures, PL spectra of ML MoSe_2_ and PtSe_2_–MoSe_2_ heterostructures were collected. As shown in Figure 1e, a pronounced peak at 1.55 eV is from ML MoSe_2_, while a weaker PL peak presents in the heterostructures. Such a photophysical phenomenon might be ascribed to the additional exciton decay channel supplied by the PtSe_2_ layer, via mechanisms of charge transfer or energy transfer, as previously reported.^[^ ^16^, ^23^ ^]^

### 2.2 Exciton Dynamics Excited Above MoSe_2_ Transition Energy

**Figure 2:**
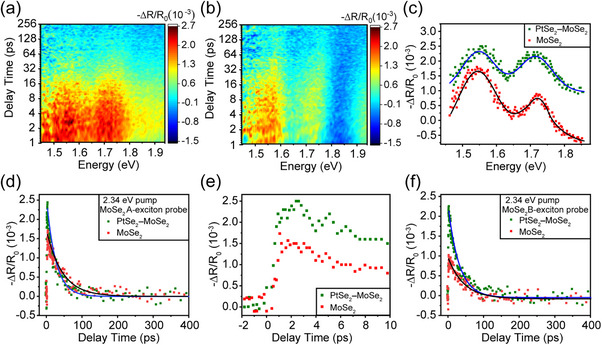
Ultrafast dynamics of PtSe_2_ **–**MoSe_2_ heterostructures and pure MoSe_2_ under the excitation at 2.34 eV. TR contour plot, a) for the heterostructure and b) pure MoSe_2_. c) TR spectra extracted from a, b) with the delay of 1.51 ps. The fitted Gaussian curves show two prominent peaks, one at the A‐exciton (1.55 eV) and the other at the B‐exciton (1.73 eV). d) TR kinetics curves of the A‐exciton resonance extracted from a, b), respectively. Solid dark‐green squares denote the PtSe_2_ **–**MoSe_2_ heterostructure, while solid red squares denote pure MoSe_2_. Both the kinetics are single‐exponentially fitted. The kinetics extracted from d) within the initial 10 ps is shown in e). f) TR kinetics curves of the B exciton resonance extracted from pure MoSe_2_ and heterostructure, respectively, fitted by a single exponential function.

The excited carrier dynamics of the heterostructures were investigated by femtosecond time‐resolved pump‐probe spectroscopy. **Figure** **2**a,b presents the 2D TR contour plots of the PtSe_2_–MoSe_2_ heterostructures and pure MoSe_2_ under the excitation with a 2.34 eV laser, with pump fluence of 87.5 µJ cm^−2^. Here, both MoSe_2_ and PtSe_2_ layers can be excited via this pulse. The TR spectra with a delay time of 1.51 ps extracted from the TR plots are shown in Figure 2c. The positive peaks located at 1.55 and 1.73 eV are attributed to photo‐induced bleaching (PIB) signals,^[^ ^8^, ^23^ ^]^ corresponding to the A and B exciton resonances of MoSe_2_, respectively. Compared with the A and B exciton resonances on a SiO_2_/Si substrate,^[^ ^16d^ ^]^ both peaks are slight redshift owing to the dielectric screening environment by BLG.^[^ ^9^ ^]^ The kinetics of the MoSe_2_ A‐exciton resonances of the PtSe_2_–MoSe_2_ heterostructures and pure MoSe_2_, with green and red squares respectively, are shown in Figure 2d, while the kinetics within the initial several picoseconds are shown in Figure 2e. Compared with pure MoSe_2_, the significantly enhanced intensities for the MoSe_2_ A‐exciton and B‐exciton in the heterostructure in Figure 2f suggest that the charge transfer between PtSe_2_ and MoSe_2_ should exists, since these two features mainly reflect the exciton dynamics in the bottom direct‐bandgap MoSe_2_ layer, not in the upper indirect‐bandgap PtSe_2_. Intriguingly, a slightly oscillatory signal occurs within the first few picoseconds in Figure 2e, which is attributed to the breathing phonon modes that arise from inter‐vibration between the interface of PtSe_2_ and MoSe_2_ layers.^[^ ^24^ ^]^ Such oscillation modulates the ΔR/R signals which should be taken into consideration in the design of PtSe_2_‐based devices.

The kinetics for A‐exciton resonances are exponentially fitted with decay lifetimes of 44.9 ± 3.6 and 26.1 ± 1.5 ps in the pure MoSe_2_ and heterostructure respectively. Similarly, the decay lifetimes for the corresponding B‐excitons are 45.4 ± 3.5 and 30.6 ± 1.4 ps respectively. These findings are consistent with the lower PL intensity observed for the heterostructure in Figure 1e. The shorter decay lifetime in PtSe_2_–MoSe_2_ heterostructure indicates that it has potential applications for sensitive optoelectronic devices. It should be noted that the signal‐to‐noise (S/N) ratio for these kinetics in Figure 2 is not particularly high, which can be attributed to the conductive BLG‐SiC (0001) substrate. More discussion about the S/N ratio in Section S3 (Figure S2, Supporting Information). The clear difference in these kinetics can gain insight into the dynamic behavior of excited states to some extent.^[^ ^23^ ^]^

### 2.3 Exciton Dynamics Excited Below MoSe_2_ Transition Energy

**Figure 3:**
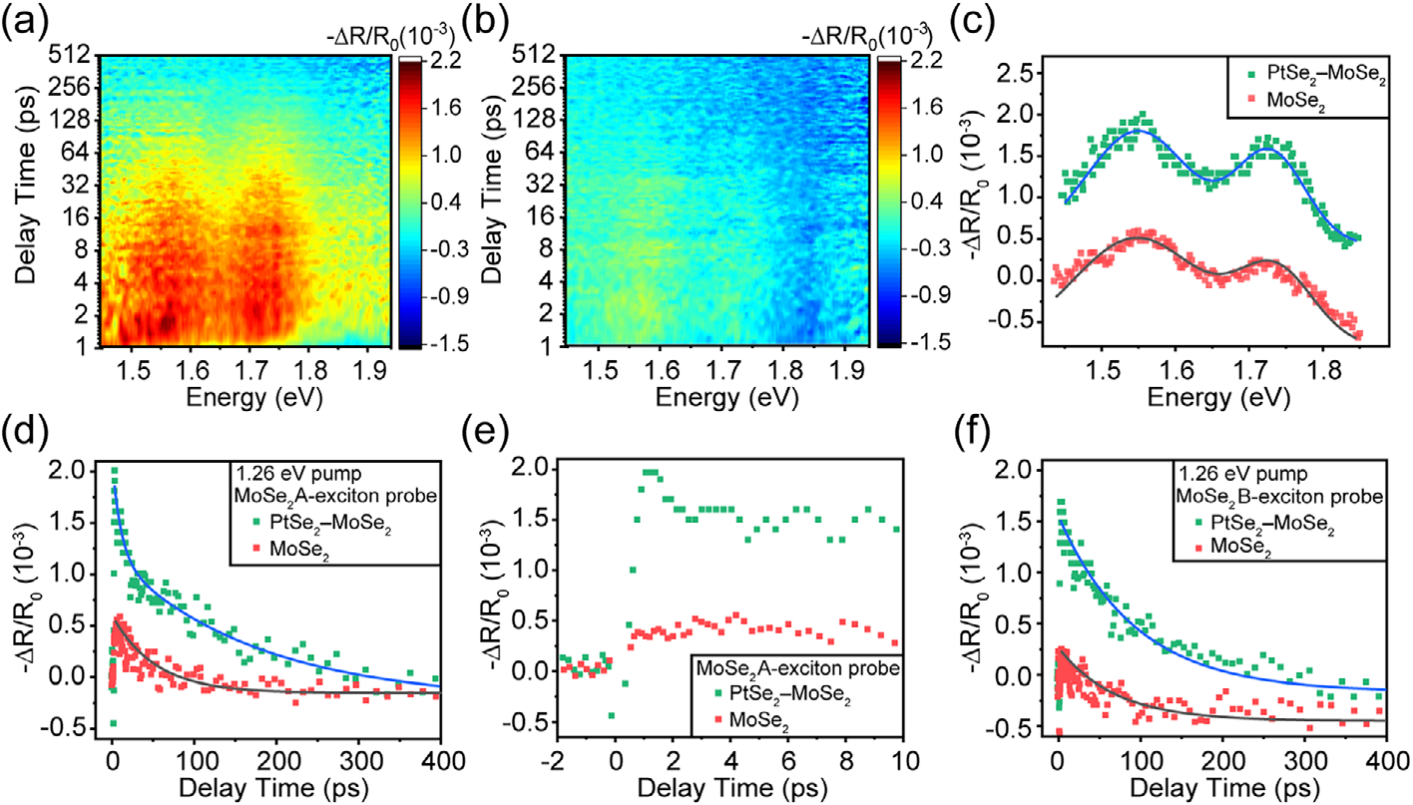
Ultrafast dynamics of PtSe_2_ **–**MoSe_2_ heterostructure and pure MoSe_2_ under the excitation at 1.26 eV. TR contour plot of a) PtSe_2_ **–**MoSe_2_ heterostructure and b) pure MoSe_2_. c) TR spectra extracted from a,b) with the delay of 1.56 ps. Two distinct peaks occur in the curves fitted by the Gaussian function. d) Kinetics of the A‐exciton resonance derived from a, b), fitted by a single exponential function. Solid light‐green squares denote the heterostructure, and light‐red squares denote pure MoSe_2_. e) The TR kinetics extracted from d) within the initial 10 ps. f) TR kinetics curves of the B exciton resonance extracted from pure MoSe_2_ and heterostructure, fitted by a single exponential function.

To further explore the carrier transfer in the heterostructure, pump‐probe spectroscopy was performed under a 980 nm (1.26 eV) laser with a pump fluence of 0.56 mJ cm^−2^. This pump energy is much lower than the optical bandgap (1.55 eV) for ML MoSe_2_, but higher than that of ML PtSe_2_ (1.13 eV).^[^ ^14^ ^]^ Thus, the single‐photon energy can only excite electrons in the valence band for the PtSe_2_ layer, but not for the MoSe_2_ layer. Nevertheless, two resonant features are similar to the PIB peaks of A and B excitons in ML MoSe_2_ in Figure 2b appear in **Figure** **3**a, although the MoSe_2_ layer was not excited by the pump laser. To rule out the hot carrier transfer from the BLG substrate,^[^ ^25^ ^]^ control experiments were performed on a pure MoSe_2_ layer on a BLG substrate under the same conditions, showing two extremely weak spectral features in Figure 3b. In Figure 3c, both the TR spectra for the PtSe_2_–MoSe_2_ heterostructure and pure MoSe_2_ at the time delay of 1.56 ps show almost identical features except for the intensities compared with those in Figure 2c, indicating that both are from the resonances of MoSe_2_. It implies that PtSe_2_–MoSe_2_ heterostructure breaks the limitation of MoSe_2_ photoresponse by the intrinsic optical bandgap, and the inter‐band transition range expands to near‐infrared wavelengths.

The kinetics of the MoSe_2_ A‐exciton resonance in PtSe_2_–MoSe_2_ heterostructure (solid light‐green squares) and pure MoSe_2_ (solid light‐red squares) are listed in Figure 3d, while those within the initial several ps are listed in Figure 3e. By exponentially fitting the kinetics for the MoSe_2_ A‐exciton, the decay lifetimes of 63.4 ± 3.9 and 92.2 ± 3.7 ps for pure MoSe_2_ and PtSe_2_–MoSe_2_ heterostructure are obtained respectively. Moreover, the kinetics for the B exciton are presented in Figure 3f, showing similar behavior as those of the A exciton. Their decay lifetimes are also fitted by the same exponential function, with the decay lifetimes of 65.8 ± 6.8 ps (pure MoSe_2_) and 93.8 ± 4.8 ps (heterostructure), respectively. These lifetimes are in contrast to those in Figure 2d excited by a 2.34 eV laser, in which the lifetime in the ML MoSe_2_ is longer than that in the heterostructure. The decay constants in the heterostructure are much longer than those in the pure MoSe_2_, which is consistent with the expected longer lifetime arising from the spatial separation of carriers at the interface.

Additionally, these kinetics indicate that TR intensities of the A‐exciton resonance in MoSe_2_ within the heterostructure are almost 4 times higher than those of isolated MoSe_2_, and the rise time for the heterostructure is nearly identical to that of the pure MoSe_2_ in Figure 3e. The enhanced intensities and the same rise time are another evidence of the interfacial carrier transfer. Without the carrier transfer process, the kinetic signal should be indistinguishable between the isolated MoSe_2_ and heterostructure. Hence, it is believed that the observed resonant features in the PtSe_2_–MoSe_2_ heterostructure are attributed to the interfacial carrier transfer from PtSe_2_ to MoSe_2_, which is the dominating factor for the carriers’ behavior in the heterostructure. Subsequently, the fluence‐dependent pump‐probe spectroscopy measurements were performed on the heterostructure with a 1.26 eV laser (Figure S3, Supporting Information). The intensities of the TR signals are proportional to the pump fluence in the range of experimental power intensities, which rules out the effect of nonlinear excitation processes, e.g., two‐photon absorption.

### 2.4 Discrepancies Between Theoretical Calculations and Transient Experiments

According to the previously calculated type‐II band alignment of the PtSe_2_–MoSe_2_ heterostructure in Figure 1c,^[^ ^16^, ^20^ ^]^ both CBM and VBM are in different layers. This band‐alignment would drive one type of photocarrier to the opposite layer while retaining the other type within this layer. It has been demonstrated that such interlayer charge separation would form interlayer excitons with long lifetimes.^[^ ^23^, ^26^ ^]^ The interlayer excitons can generate quite robust and long‐lived transient reflection features at the intralayer exciton resonance of a given layer since the exciton states are superimposed by the conduction band (CB) and valence band (VB) states. In this case, the exciton signal from MoSe_2_ in heterostructure should be stronger and longer‐lived than that of pure MoSe_2_. However, the decay lifetime of the exciton of MoSe_2_ in heterojunction is shorter than that of pure MoSe_2_ under 2.34 eV excitation, which is inconsistent with the features dominated by the type‐II alignment. Therefore, further studies have to be carried out to reveal the accurate energy band alignments of PtSe_2_–MoSe_2_ heterostructures grown on the given conductive substrate, to give the precise interpretation for the above carrier dynamics.

### 2.5 Interfacial Atomic Structure and Electronic Properties

**Figure 4:**
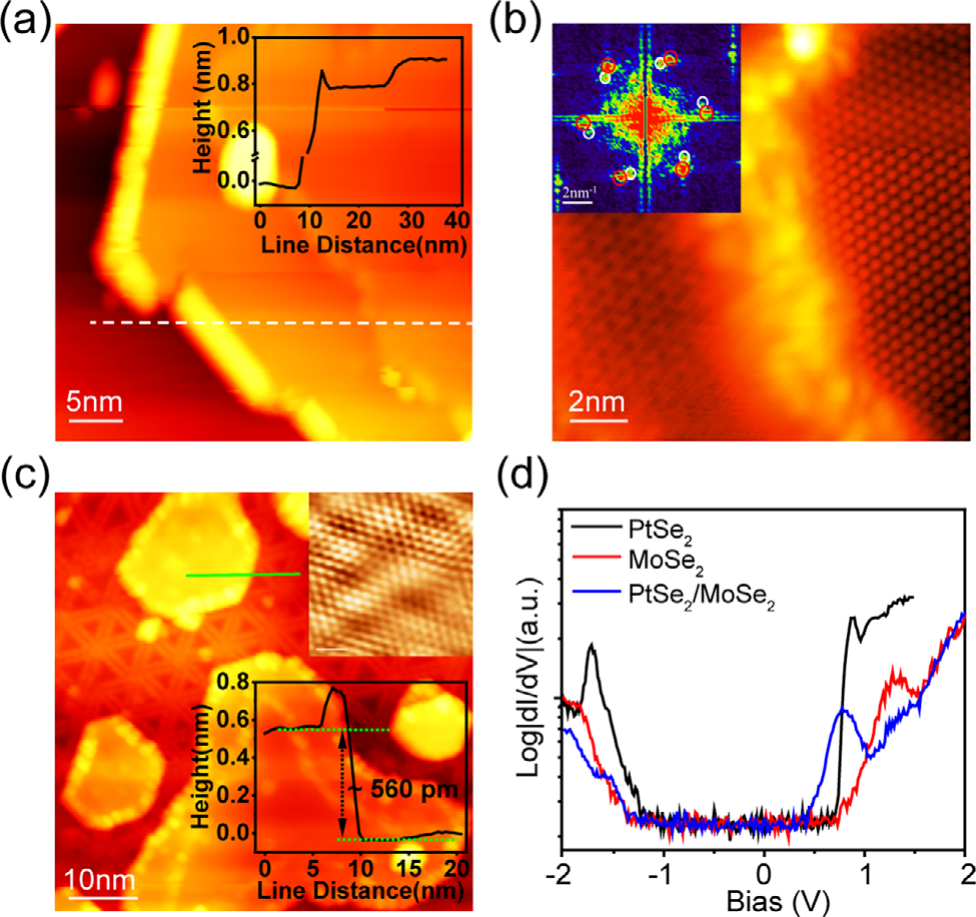
STM characterization of PtSe_2_ **–**MoSe_2_ heterostructure. a) A STM topographic image of a PtSe_2_ **–**MoSe_2_ lateral heterojunction. The inset shows the height profile of the surface among the white dashed line across substrate, PtSe_2_, and MoSe_2_. V_b_ = 2.5 V, I_t_ = 20 pA. b) An atomic‐resolution STM image showing the lateral junction between ML PtSe_2_ (left) and ML MoSe_2_ (right). An FT‐STM pattern in the inset shows the reciprocal lattice of PtSe_2_ and MoSe_2_, as highlighted by the white and red circles, respectively. V_b_ = −1 V, I_t_ = 180 pA. c) A STM topography image for a PtSe_2_/MoSe_2_ vertical heterojunction. Top and bottom layers represent PtSe_2_ and MoSe_2_, respectively. The upper inset shows the atomic image of the PtSe_2_ surface in vertical heterojunction processed by a Fast Fourier Transform filter, the raw image is in Figure S6d (Supporting Information). Scale bar: 1.2 nm. And the lower inset presents a height profile along the green solid line. V_b_ = −1.8 V, I_t_ = 25 pA. d) STS spectra for ML PtSe_2_ on ML MoSe_2_ (blue), ML MoSe_2_ (red) and ML PtSe_2_ (black), taken from adjacent domains but far from the interface region. V_b_ = 1 V, I_t_ = 100 pA.

To further reveal the mechanism for the interfacial carrier transfer in PtSe_2_–MoSe_2_ heterostructures more precisely, their atomic structures and electronic properties at the interface were studied using STM/STS. STM measurements were performed on a lateral ML PtSe_2_–MoSe_2_ heterojunction, in which the in‐plane growth of ML PtSe_2_ along the edge of ML MoSe_2_, as shown in **Figure** **4**a. Approximately, the area ratio of the lateral is 36% of the total heterojunction (Figure S4, Supporting Information). The inset line profile demonstrates that the height difference between the PtSe_2_ and MoSe_2_ is ≈0.1 nm, which was further confirmed by the line profiles collected on the isolated ML PtSe_2_ and ML MoSe_2_, respectively (Figure S5, Supporting Information). Such height difference illustrates the difference in structure between the sides of the interface.

An atomic‐resolution STM image of the lateral heterojunction shows two different atomic structures separated by a buffer layer in Figure 4b, in which the left and right sides correspond to PtSe_2_ and MoSe_2,_ respectively. Unlike other lateral heterojunctions with a relatively sharp interface,^[^ ^6^, ^7^, ^27^ ^]^ the interface between PtSe_2_ and MoSe_2_ is messy, which may be attributed to the different lattice symmetries and large lattice parameter difference. 1H phase MoSe_2_ has the symmetry of $P_{\bar{6}m2}$ with in‐plane lattice parameters of 3.28 Å, while 1T phase PtSe_2_ belongs to the space group of $P_{\bar{3}m1}$ with in‐plane lattice parameters of 3.73 Å. The large lattice mismatch of 13% was confirmed by the RHEED measurements in Figure 1a. Besides, the lattice rotation between the MoSe_2_ and epitaxial PtSe_2_ was demonstrated by the Fourier Transform of STM (FT‐STM) pattern in the inset in Figure 4b and Figure S6 (Supporting Information). To decrease the strain caused by the lattice mismatch and rotation, a buffer layer with distorted structures occurs between ML MoSe_2_ and PtSe_2_, which brings abundant localized electronic states, making it difficult to obtain atomic structures by STM (Section S8, Supporting Information).

Moreover, vertical PtSe_2_/MoSe_2_ heterojunctions were also observed on the same substrate, where ML PtSe_2_ stays on a MoSe_2_ monolayer, as shown in Figure 4c. The characteristic wagon‐wheel‐like patterns formed by inversion domain boundary defects are seen in the bottom layer, due to 1D Se defects.^[^ ^21^, ^28^ ^]^ The upper inset shows the atomic structures of PtSe_2_ atop the heterojunction, while the hetero‐bilayer shows a certain corrugation due to the lattice mismatch (13%) and twist angle (Figure S6h, Supporting Information). While the lower‐right line profile along the green dashed line reveals the step height of ≈0.56 nm, resembling the height difference between adjacent PtSe_2_ layers.^[^ ^13^, ^29^ ^]^ The apparent height variations observed in STM topographic measurements originate from the geometric heights and electronic states. When compared to ML PtSe_2_(≈0.8 nm) on an epitaxial graphene substrate, the height difference could be attributed to the difference in electron injection into the PtSe_2_ layer from graphene versus the underlying MoSe_2_, as well as the distinct interfacial van der Waals coupling strengths. The STS spectra were taken on the adjacent PtSe_2_ and MoSe_2_ monolayer domains far from the interface regions, as well as the top layer of the vertical heterostructure, as shown in Figure 4d. The deduced electronic bandgaps were shown to be 1.87 ± 0.03 eV for ML PtSe_2_ and 2.19 ± 0.04 eV for ML MoSe_2_, consistent with previously reported.^[^ ^9^, ^13^, ^30^ ^]^ Besides, the bandgap for the vertical heterostructures is 1.69 ± 0.02 eV. However, the interaction of the local electric field between the tip and sample surface might introduce certain errors to the bandgap measurement.^[^ ^31^ ^]^ (More discussion of tip‐induced band bending in Section S9, Supporting Information)

### 2.6 Both Interfaces and Two Different Band Alignments

**Figure 5:**
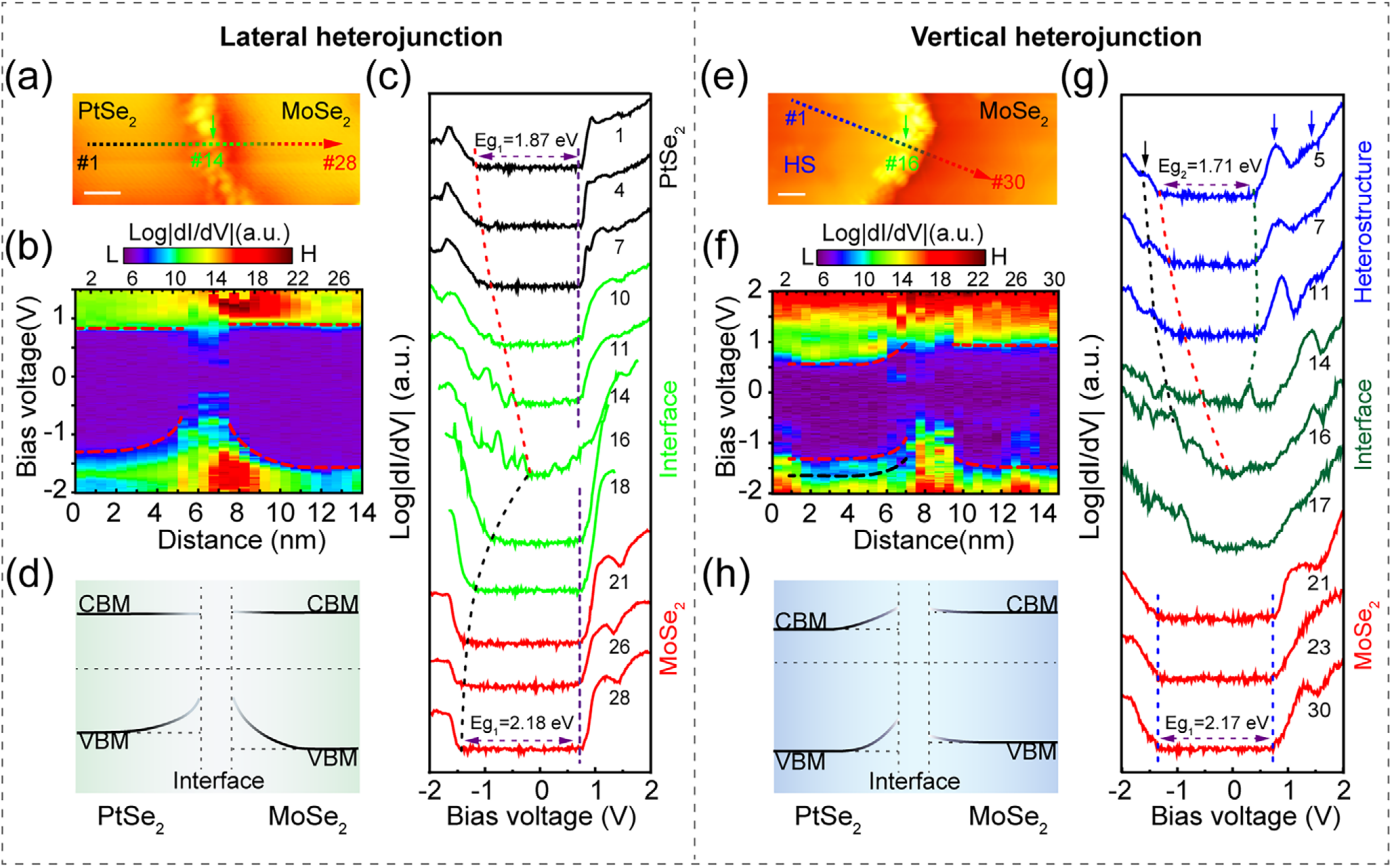
Electronic structures evolution across the lateral and vertical PtSe_2_–MoSe_2_ heterojunction interface. An STM image of the a) lateral heterojunction and e) vertical heterojunction. V_b_ = 1 V, I_t_ = 100 pA. Scale bar: 2 nm. b, f) The spatial dependence of constant height dI/dV spectra were taken along the colorful dashed line with a guide arrow in a, e), respectively. b) The total length for the collection is ≈14 nm with a step increment of 0.5 nm, Spectrum #14 was taken right at the lateral heterojunction interface. f) The total length of the path is 15 nm with a step increment of 0.5 nm. Spectrum #16, exhibiting the narrowest bandgap, was taken near the interface of the vertical heterojunction, while spectrum #20 represents the onset of underlying MoSe_2_. The locations of the energy levels at the band edges are marked by red dashed lines. c, g) Selected log (dI/dV) spectra from b, f). The colorful dashed line indicates the spatially dependent energy band bending effect. In c), the spectra taken on ML PtSe_2_, interface, and ML MoSe_2_ are shown with black, green, and red lines, respectively. The bandgaps of ML MoSe_2_ and ML PtSe_2_ at the position far away from the interface are indicated by the violet. In g), the spectra taken on vertical heterojunction, interface, and ML MoSe_2_ are shown by the blue, dark‐green, and red curves, respectively. V_b_ = 1.0 V, I_t_ = 100 pA. d, h) Energy level diagram of the lateral and vertical PtSe_2_–MoSe_2_ heterojunctions interface, based on the line scans dI/dV spectra.

To investigate the band alignment at the interface of lateral and vertical PtSe_2_–MoSe_2_ heterojunctions in detail, STS line scans were taken along the colorful dashed line with a guide arrow in **Figure** **5**a,e, respectively. Contour plots of collected STS curves are demonstrated in Figure 5b,f, and selected individual spectra are shown in Figure 5c,g, respectively. The results unveil a distinct difference in the band alignment between the lateral and vertical heterojunction interfaces. The apparent band bending for the VB and CB is near the heterojunction interface, as guided with red dashed lines in Figure 5b,f. Notably, for lateral heterojunction, both CBs on two sides of the interface are almost aligned. For vertical heterojunction, the band bending mainly occurs only in the heterojunction region to the left of the interface. Approaching the interfacial region, mid‐gap states are clearly seen in Figure 5b,f. The localized electrons (holes) produce a built‐in electric field in the space charge region, which would contribute to the band bending near the interface. The interface states with a narrow bandgap can be deemed as 1D electronic system, which might have potential applications in controlling carrier transport. (More detailed discussions about the interfacial states and band bending in Section S10, Supporting Information)

On the one hand, in the area far away from the interface of lateral heterojunction, the CBM of the right MoSe_2_ is only slightly upshifted (0.02 eV) relative to that of the left PtSe_2_, while the VBM of PtSe_2_ and MoSe_2_ are offset by more than 0.3 eV in Figure 5c. This energy level alignment is contrary to the calculated work functions for individual MoSe_2_ and PtSe_2_ monolayers,^[^ ^32^ ^]^ which can be attributed to the Fermi energy level pinning effect from the substrate.^[^ ^33^ ^]^ The DFT calculation results showed that CBM and VBM energy levels for ML MoSe_2_ are at −3.98 and −5.49 eV,^[^ ^16d^ ^]^ similar to the photoemission experiment results of −3.8 and −5.98 eV,^[^ ^34^ ^]^ respectively. As for ML PtSe_2_, the CBM values were calculated to be from −4.29 to −4.56 eV, and the VBM values range from −5.49 to −5.9 eV.^[^ ^16^, ^35^ ^]^ From the above‐reported energy levels, the heterojunction would form a type‐II band alignment. Nevertheless, when the MoSe_2_ and PtSe_2_ are grown on a semi‐metallic graphite substrate, their Fermi levels will be pinned by the substrate, altering the band alignment of the CBM and VBM. Hence, the PtSe_2_–MoSe_2_ lateral heterojunction exhibits type‐I band alignment with a slight difference at the CBM, as shown in the schematic simplified energy level in Figure 5d.

On the other hand, from the STS spectra far from the vertical heterojunction interface (spectrum #30 in Figure 5g), it can be noted that the energy levels of both the CBM and VBM for the bottom MoSe_2_ are consistent with those in Figure 4d. Second, the energy level of CBM for the top PtSe_2_ shifts toward the Fermi energy level due to its isolation from the substrate (as indicated by the green dashed line in Figure 5g), while the energy level of VBM for the heterostructures is aligned with that for MoSe_2_ owing to the tunneling from the bottom MoSe_2_ layer. Considering the electronic bandgap of ML PtSe_2_, the VBM of the top layer PtSe_2_ should be located at the peak with the black arrow (as indicated by the black dashed line in Figure 5g). Such alignments between the top PtSe_2_ and bottom MoSe_2_ indicate that the vertical heterojunction is a type‐II band alignment as schematically depicted in Figure 5h, agreeing with the calculated result.^[^ ^16^, ^20^ ^]^ Besides, two shoulder peaks at 0.9 and 1.4 eV in the CB marked by the blue arrows could be ascribed to the coupling between PtSe_2_ and MoSe_2_.

### 2.7 Bipolar Charge Transfer Mechanism

Even more importantly, the electronic bandgap (E_g_) deduced from the STS spectrum (Figure 4d) is much larger than the optical bandgap (E_opt_) from both PL and TR spectrum (Figures 1e and 2c). The difference between these energies is attributed to the giant exciton binding energy (E_g_‐E_opt_) of monolayer selenides.^[^ ^9^ ^]^ Since the exciton binding energies for the monolayer selenides are nearly the same,^[^ ^11^ ^]^ the energy band alignment deduced from STS spectra should be nearly the same as that for optical excitons. Therefore, based on the band alignments from STM/STS measurements, the interfacial carrier transfer in the PtSe_2_–MoSe_2_ heterostructure is well interpreted.

**Figure 6:**
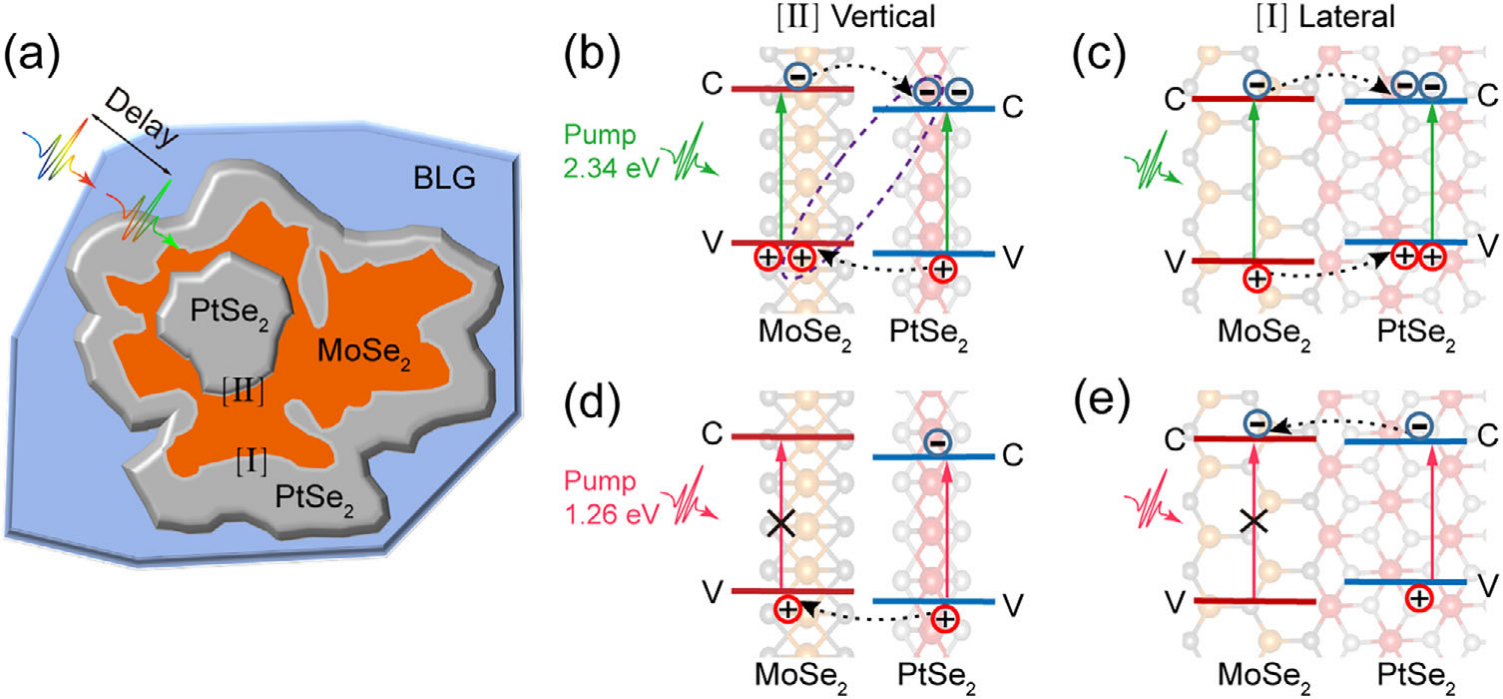
Schematic illustration of the charge transfer process in the PtSe_2_ **–**MoSe_2_ heterostructure. a) Schematic diagram of the different interface configurations in PtSe_2_ **–**MoSe_2_ heterostructure. b) Schematic energy band diagrams of the PtSe_2_/MoSe_2_ vertical heterostructure with the excitation of a 2.34 eV laser, showing that charge transfer in heterostructure. Both layers of the heterostructure are excited by the 2.34 eV laser, with electrons (‐) and holes (+) populating the conduction band minimum (C, CBM) and valence band maximum (V, VBM). c) Energy level diagram and the interfacial charge transfer process in the PtSe_2_ **–**MoSe_2_ lateral heterostructure with a 2.34 eV laser, which would drive the ambipolar move to the opposite side. d) Diagram for the holes transfer from PtSe_2_ to MoSe_2_ in vertical heterostructure with the excitation of a 1.26 eV laser, where the single‐photon energy can only excite electrons in the VBM for the PtSe_2_ layer. e) Schematic illustration of the interfacial electron transfer from PtSe_2_ to MoSe_2_ in the lateral heterostructure under the excitation by a 1.26 eV laser.

It contains two interface configurations as sketched in **Figure** **6**a, namely [I] lateral and [II] vertical PtSe_2_–MoSe_2_ heterojunction, which also corresponds to the type‐I and type‐II band alignments, respectively. The above STS measurements show that, in general, the energy level of the CBM for MoSe_2_ is higher than that for PtSe_2_ and the energy level of VBM for PtSe_2_ is lower than that of MoSe_2_ in the type‐II vertical heterojunction. Hence, after the excitation of a 2.34 eV laser, excited electrons in the CBM for MoSe_2_ will hop into the CBM for PtSe_2_ in the heterostructure, while the holes in the VBM for PtSe_2_ can emigrate into the VBM for MoSe_2_, as schematically shown in Figure 6b. The spare electrons in the CBM for PtSe_2_ and holes in the VBM for MoSe_2_ form indirect excitons due to the rotation angle between PtSe_2_ and MoSe_2_ revealed by FT‐STM patterns (Figure S6h, Supporting Information). Such electrons and holes hopping between MoSe_2_ and PtSe_2_ are ultrafast (less than 1 ps),^[^ ^36^ ^]^ but the recombination of indirect interlayer excitons is significantly slower than the former process, resulting in net carrier accumulation in MoSe_2_, which in turn increases the intensities of PIB peaks (Figure 2e).

Intriguingly, the existence of type‐I band alignment arises from the Fermi energy levels pinned by the substrate in lateral heterojunction. After excitation, with both electrons and holes transfer from MoSe_2_ to PtSe_2_ (Figure 6c), such ambipolar transfer leads to the depopulation of the exciton, which further weakens the PL intensities for the MoSe_2_ in heterostructure (Figure 1e). Previous studies have shown that the decay of excitons in a given layer in type‐I heterojunction has a shorter or similar time constant compared to the intrinsic one, due to the formation of the additional decay channel.^[^ ^2^, ^37^ ^]^ The lack of a slow decay lifetime in heterostructure indicates that both electrons and holes are transferred. This explains the shorter decay constant of MoSe_2_ excitons in heterostructure than pure MoSe_2_ at 2.34 eV excitation. Thus, despite the relatively little area occupation of the lateral heterojunction, the impact of exciton dynamic derived from the type‐I band alignment due to Fermi energy levels pinned by the substrate is not negligible.

Moreover, upon the excitation of a 1.26 eV laser, only electrons in the VBM of PtSe_2_ in the heterostructure were excited, and the electrons in the MoSe_2_ layer cannot be excited. For type‐II vertical heterojunction, the excited holes in the VBM for the PtSe_2_ in heterostructure hop into the VBM for the MoSe_2_ (Figure 6d), strongly increasing the PIB peak corresponding to the A‐exciton resonances in Figure 3a. Besides, in the type‐I lateral heterojunction, the excited electrons in the CBM of the PtSe_2_ monolayer can also hop into the CBM for the MoSe_2_ due to the nearly same energy level, as schematically shown in Figure 6e. Most of the previous studied TMD‐based heterojunctions are H‐phase crystal structures. However, in this work, the heterostructure comprises 1T‐PtSe_2_ and 1H‐MoSe_2_, and the different lattice structure introduces robust interfacial interaction (as evidenced by the dI/dV spectra in Figure 5b,f), affecting the coherent charge transfer process. Second, due to the width of the lateral junction, the charge displacement would exceed that observed in the vertical. Integrating the above analysis, when the unidirectional photogenerated carriers hopping from PtSe_2_ to MoSe_2_ in whether lateral or vertical heterojunction, the decay lifetimes are even longer than that excited at 2.34 eV.

## 3 Conclusion

In summary, the carrier dynamics of MBE‐grown atomically thin PtSe_2_–MoSe_2_ heterostructures have been studied, and the corresponding mechanism was interpreted by the STM/STS measurements. Under the excitation of a 2.34 eV femtosecond laser, the ultrafast transient spectra demonstrated that interfacial carrier transfer occurred between MoSe_2_ and PtSe_2_, which was further confirmed by similar measurements under a 1.26 eV femtosecond laser. Lateral and vertical heterojunctions were found to exist between 1T‐PtSe_2_ and 1H‐MoSe_2_ through atomic STM images, which are identified to be the type‐I and type‐II band alignment by the line scan STS spectra, respectively. These two types of band alignments can give a well interpretation of the transient spectra under different excitation wavelengths, demonstrating the different charge transfer at the interface of the hetero‐phase heterojunction. Notably, the influence of the substrate on carrier dynamics at the heterojunction interface is revealed. Our work provides a deeper comprehension of the electronic structures and carrier dynamics of PtSe_2_–MoSe_2_ heterojunctions with ultrahigh spatial and temporal resolutions. Besides, as a typical work on monolayer heterostructures by the combination of STM/STS and ultrafast pump‐probe spectroscopy, these measurements with high spatial and temporal resolutions can be extended to interfacial studies on other semiconductor heterostructures.

## Conflict of Interest

The authors declare no conflict of interest.

## Supporting information



Supporting Information

## Data Availability

The data that support the findings of this study are available from the corresponding author upon reasonable request.

## References

[1] a) Y. Liu , N. O. Weiss , X. Duan , H.‐C. Cheng , Y. Huang , X. Duan , Nat. Rev. Mater. 2016, 1, 16042;

[2] a) D. B. Sulas‐Kern , E. M. Miller , J. L. Blackburn , Energy Environ. Sci. 2020, 13, 2684;

[3] a) V. R. Policht , M. Russo , F. Liu , C. Trovatello , M. Maiuri , Y. Bai , X. Zhu , S. Dal Conte , G. Cerullo , Nano Lett. 2021, 21, 4738; 34037406 10.1021/acs.nanolett.1c01098 PMC8289282

[4] a) C. Xia , B. Xue , T. Wang , Y. Peng , Y. Jia , Appl. Phys. Lett. 2015, 107, 193107;

[5] a) Z. Shi , X. Wang , Y. Sun , Y. Li , L. Zhang , Semicond. Sci. Technol. 2018, 33, 093001;

[6] a) J. Park , J. Lee , L. Liu , K. W. Clark , C. Durand , C. Park , B. G. Sumpter , A. P. Baddorf , A. Mohsin , M. Yoon , G. Gu , A. P. Li , Nat. Commun. 2014, 5, 5403; 25377633 10.1038/ncomms6403

[7] a) M. H. Chiu , C. Zhang , H. W. Shiu , C. P. Chuu , C. H. Chen , C. Y. Chang , C. H. Chen , M. Y. Chou , C. K. Shih , L. J. Li , Nat. Commun. 2015, 6, 7666; 26179885 10.1038/ncomms8666 PMC4518320

[8] J. Peng , D. Yang , C. Ren , Y. Jiang , X. Zhu , F. Jing , H. Qiu , H. Liu , Z. Hu , Adv. Mater. 2022, 34, 2107738. 10.1002/adma.202107738 34989034

[9] M. M. Ugeda , A. J. Bradley , S. F. Shi , F. H. da Jornada , Y. Zhang , D. Y. Qiu , W. Ruan , S. K. Mo , Z. Hussain , Z. X. Shen , F. Wang , S. G. Louie , M. F. Crommie , Nat. Mater. 2014, 13, 1091. 25173579 10.1038/nmat4061

[10] M. Bernardi , M. Palummo , J. C. Grossman , Nano Lett. 2013, 13, 3664. 23750910 10.1021/nl401544y

[11] H. J. Liu , L. Jiao , L. Xie , F. Yang , J. L. Chen , W. K. Ho , C. L. Gao , J. F. Jia , X. D. Cui , M. H. Xie , 2D Mater. 2015, 2, 034004.

[12] a) K. Wang , B. Huang , M. Tian , F. Ceballos , M. W. Lin , M. Mahjouri‐Samani , A. Boulesbaa , A. A. Puretzky , C. M. Rouleau , M. Yoon , H. Zhao , K. Xiao , G. Duscher , D. B. Geohegan , ACS Nano 2016, 10, 6612; 27309275 10.1021/acsnano.6b01486

[13] a) J. Li , S. Kolekar , M. Ghorbani‐Asl , T. Lehnert , J. Biskupek , U. Kaiser , A. V. Krasheninnikov , M. Batzill , ACS Nano 2021, 15, 13249; 10.1039/d0nr08345c 33393546

[14] Y. Zhao , J. Qiao , Z. Yu , P. Yu , K. Xu , S. P. Lau , W. Zhou , Z. Liu , X. Wang , W. Ji , Y. Chai , Adv. Mater. 2017, 29, 1604230. 10.1002/adma.201604230 27886410

[15] G. Wang , Z. Wang , N. McEvoy , P. Fan , W. J. Blau , Adv. Mater. 2021, 33, 2004070. 10.1002/adma.202004070 33225525

[16] a) J. Yuan , T. Sun , Z. Hu , W. Yu , W. Ma , K. Zhang , B. Sun , S. P. Lau , Q. Bao , S. Lin , S. Li , ACS Appl. Mater. Interfaces 2018, 10, 40614; 30387989 10.1021/acsami.8b13620

[17] H.‐S. Kim , J. Jeong , G.‐H. Kwon , H. Kwon , M. Baik , M.‐H. Cho , Appl. Surf. Sci. 2022, 585, 152507.

[18] a) P. Wang , D. He , Y. Wang , X. Zhang , X. He , J. He , H. Zhao , ACS Appl. Mater. Interfaces 2021, 13, 57822; 34797636 10.1021/acsami.1c18189

[19] L.‐H. Zeng , S.‐H. Lin , Z.‐J. Li , Z.‐X. Zhang , T.‐F. Zhang , C. Xie , C.‐H. Mak , Y. Chai , S. P. Lau , L.‐B. Luo , Y. H. Tsang , Adv. Funct. Mater. 2018, 28, 1705970.

[20] L. Xiang , Y. Ke , Q. Zhang , Appl. Phys. Lett. 2019, 115, 203501.

[21] a) Y. Wang , L. Li , W. Yao , S. Song , J. T. Sun , J. Pan , X. Ren , C. Li , E. Okunishi , Y. Q. Wang , E. Wang , Y. Shao , Y. Y. Zhang , H. T. Yang , E. F. Schwier , H. Iwasawa , K. Shimada , M. Taniguchi , Z. Cheng , S. Zhou , S. Du , S. J. Pennycook , S. T. Pantelides , H. J. Gao , Nano Lett. 2015, 15, 4013; 25996311 10.1021/acs.nanolett.5b00964

[22] S. M. Poh , S. J. Tan , X. Zhao , Z. Chen , I. Abdelwahab , D. Fu , H. Xu , Y. Bao , W. Zhou , K. P. Loh , Adv. Mater. 2017, 29, 1605641. 10.1002/adma.201605641 28112835

[23] X. Hong , J. Kim , S. F. Shi , Y. Zhang , C. Jin , Y. Sun , S. Tongay , J. Wu , Y. Zhang , F. Wang , Nat. Nanotechnol. 2014, 9, 682. 25150718 10.1038/nnano.2014.167

[24] X. Chen , S. Zhang , L. Wang , Y.‐F. Huang , H. Liu , J. Huang , N. Dong , W. Liu , I. M. Kislyakov , J. M. Nunzi , L. Zhang , J. Wang , Photonics Res. 2019, 7, 1416.

[25] a) M. Massicotte , P. Schmidt , F. Vialla , K. Watanabe , T. Taniguchi , K. J. Tielrooij , F. H. L. Koppens , Nat. Commun. 2016, 7, 12174; 27412308 10.1038/ncomms12174 PMC4947168

[26] F. Ceballos , M. Z. Bellus , H. Y. Chiu , H. Zhao , ACS Nano 2014, 8, 12717. 25402669 10.1021/nn505736z

[27] C. Herbig , C. Zhang , F. Mujid , S. Xie , Z. Pedramrazi , J. Park , M. F. Crommie , Nano Lett. 2021, 21, 2363. 33719457 10.1021/acs.nanolett.0c04204

[28] H. Liu , H. Zheng , F. Yang , L. Jiao , J. Chen , W. Ho , C. Gao , J. Jia , M. Xie , ACS Nano 2015, 9, 6619. 26051223 10.1021/acsnano.5b02789

[29] X. Wu , J. Qiao , L. Liu , Y. Shao , Z. Liu , L. Li , Z. Zhu , C. Wang , Z. Hu , W. Ji , Y. Wang , H. Gao , Nano Res. 2020, 14, 1390.

[30] A. J. Bradley , M. M. Ugeda , F. H. da Jornada , D. Y. Qiu , W. Ruan , Y. Zhang , S. Wickenburg , A. Riss , J. Lu , S. K. Mo , Z. Hussain , Z. X. Shen , S. G. Louie , M. F. Crommie , Nano Lett. 2015, 15, 2594. 25775022 10.1021/acs.nanolett.5b00160 PMC4415042

[31] R. M. Feenstra , J. A. Stroscio , J. Vac. Sci. Technol. B 1987, 5, 923.

[32] a) C. Gong , H. Zhang , W. Wang , L. Colombo , R. M. Wallace , K. Cho , Appl. Phys. Lett. 2013, 103, 053513;

[33] C. Zhang , Y. Chen , J. K. Huang , X. Wu , L. J. Li , W. Yao , J. Tersoff , C. K. Shih , Nat. Commun. 2016, 6, 10349. 26778119 10.1038/ncomms10349 PMC4735610

[34] J. Xiao , L. Zhang , H. Zhou , Z. Shao , J. Liu , Y. Zhao , Y. Li , X. Liu , H. Xie , Y. Gao , J. T. Sun , A. T. S. Wee , H. Huang , ACS Appl. Mater. Interfaces 2020, 12, 32099. 32603081 10.1021/acsami.0c04985

[35] S. Sattar , U. Schwingenschlogl , ACS Appl. Mater. Interfaces 2017, 9, 15809. 28443652 10.1021/acsami.7b00012

[36] H. Zhou , C. Sun , W. Xin , Y. Li , Y. Chen , H. Zhu , Nano Lett. 2022, 22, 2547. 35285224 10.1021/acs.nanolett.2c00479

[37] a) M. Z. Bellus , M. Mahjouri‐Samani , S. D. Lane , A. D. Oyedele , X. Li , A. A. Puretzky , D. Geohegan , K. Xiao , H. Zhao , ACS Nano 2018, 12, 7086; 29906088 10.1021/acsnano.8b02843

