# Supplementary material for: Unraveling the Interfacial Carrier Behavior in PtSe2–MoSe2 Heterostructures: Insights from Combined Pump‐Probe Spectroscopy and Scanning Tunneling Microscopy
Source: Adv Sci (Weinh). 2025 Mar 7;12(17):2500598. doi: 10.1002/advs.202500598 (PMC12061312; doi:10.1002/advs.202500598)
Supplement: Supplementary file 1 — Supporting Information [file ADVS-12-2500598-s001.docx]

**Supporting Information**

# Unraveling the Interfacial Carrier Behavior in PtSe_2_–MoSe_2_ Heterostructures: Insights from Combined Pump-Probe Spectroscopy and Scanning Tunneling Microscopy

Weili Zhang, Hu Chen, Jiangbo Peng, Xiaoguang Pan, Hangxin Bai, Fangli Jing, Hailong Qiu, Zhanggui Hu, Yicheng Wu, and Hongjun Liu*

Tianjin Key Laboratory of Functional Crystal Materials, Institute of Functional Crystals, School of Materials Science and Engineering, Tianjin University of Technology Tianjin 300384, China

*Corresponding email: hjliu@email.tjut.edu.cn

**Outlines:**

Section S1. Methods

Section S2. XPS measurements of PtSe_2_**–**MoSe_2_ heterostructures

Section S3. The control TR experiment for sample on the SiO_2_ substrate

Section S4. Additional data for TR measurements

Section S5. Topographic characterization of MoSe_2_ and PtSe_2_**–**MoSe_2_ heterostructure on a BLG-SiC (0001) substrate

Section S6. Thickness characterization of PtSe_2_ and MoSe_2_ monolayers on a HOPG substrate

Section S7. STM and corresponding FT-STM images of the heterostructures and TMD monolayers

Section S8. STM images of the lateral heterostructure interface

Section S9. The effect of tip induced band bending (TIBB)

Section S10. The interfacial states and band bending for the vertical heterojunction

## Section S1. Methods

**Sample Growth**

The PtSe_2_**–**MoSe_2_ heterostructures were grown by MBE in an ultra-high vacuum cavity with the pressure of 1×10^-9^ mbar. Se Flux was evaporated from the Knudsen cell, while fluxes of metals (Mo and Pt) were generated from the electron beam evaporator. The deposition rate was controlled by the flux with the ratio of Se/metal was larger than 20:1. Samples were grown on highly oriented pyrolytic graphite and bilayer graphene over 6H-SiC (0001), intended for STM testing and optical testing, respectively. For the epitaxial growth of bilayer graphene, firstly by annealing at 900 K for 12h, then rapidly increasing the temperature to 1100 K and 1700 K in sequence, holding for 10 min and 30 min, respectively, and immediately stopping the heater after the growth. During growth, the substrate was first kept at 650 K to grow MoSe_2_. The growth process was monitored by RHEED operated at 15 keV. During the growth, the former stripes gradually vanished and were taken over by a new set of weak stripes, showing the evolution of epitaxial growth. After growing the monolayer MoSe_2_, the Mo source shutter was closed while keeping the Se source continuously on, and holding for 10 min. Then, monolayer PtSe_2_ was grown by opening the Pt source shutter after decrease growth temperature to 500 K. After the growth of heterojunctions, all shutters for sources were closed. And then the grown sample was cooled down to room temperature gradually.

**Structural and Optical Characterization**

Raman and PL spectra were measured by a WITec Alfa-300 commercial confocal microscope, with an excitation laser of 2.33 eV. The continuous laser was focused on the sample with a 50× objective lens. The signal was collected by a charge-coupled device (CCD) camera.

**Ultrafast Pump-Probe Measurement**

Femtosecond Transient reflection measurements were carried by our home-built pump-probe spectroscopy system. The 1.2 eV pulse was generated from a Yb:KGW laser (Pharos, Light Conversion Ltd) with a pulse of 120 fs, 100 kHz. The fundamental laser beam of 1.2 eV was split into two beams. One beam directly went through the optical parametric amplifier (OPA) to generate the pump pulse (2.34 eV or 1.26 eV). The other beam first passed through the delay line and then was focused on the yttrium aluminum garnet crystal (YAG), yielding super-continuous white light for probing. Both the pump pulse and continuum probe pulse were collinearly focused on the sample via a short-working-distance 60× objective lens for 2.34 eV, while using a 100× objective at a long-working-distance for 1.26 eV. The reflection spectra were collected by the complementary metal oxide semiconductor (CMOS). The TR signal was calculated by the following formula: Δ*R*/*R* = (*R_on_* – *R_off_*)/*R_off_*, where *R_on_* and *R_off_* are the probe reflections with and without the pump excitation.

**STM/STS Characterization**

STM/STS experiments were carried out at 78 K in a Unisoku 1400 STM system with a DC electrochemically etched tungsten tip. All measurements were performed in constant current mode and STS measurements were taken using a phase-locked technique with a modulated voltage of 30 mV and a frequency of 978 Hz. All the applied bias was sample bias.

## Section S2. XPS measurements of PtSe_2_–MoSe_2_ heterostructures

There are two peaks in Figure S1a around 54.8 and 55.7 eV that are related to the Se 3d^5/2^ and Se 3d^3/2^, respectively. And the Pt 4f spectrum in Figure S1b can be deconvoluted into two peaks, Pt 4f^7/2^ (73.6 eV) and Pt 4f^5/2^ (76.9 eV), whereas the binding energies for the Mo 3d^5/2^ and Mo 3d^3/2^ are located at 229.4 and 232.5 eV in Figure S1c. In these charts, the energy splitting due to the spin–orbit coupling for Pt 4f (4f^7/2^ and 4f^5/2^) and Mo 3d (3d^5/2^ and 3d^3/2^) doublets change a little after forming heterostructures. An extremely slight blue shifts are observed in the binding energy of core levels in PtSe_2_**–**MoSe_2_ heterostructure.


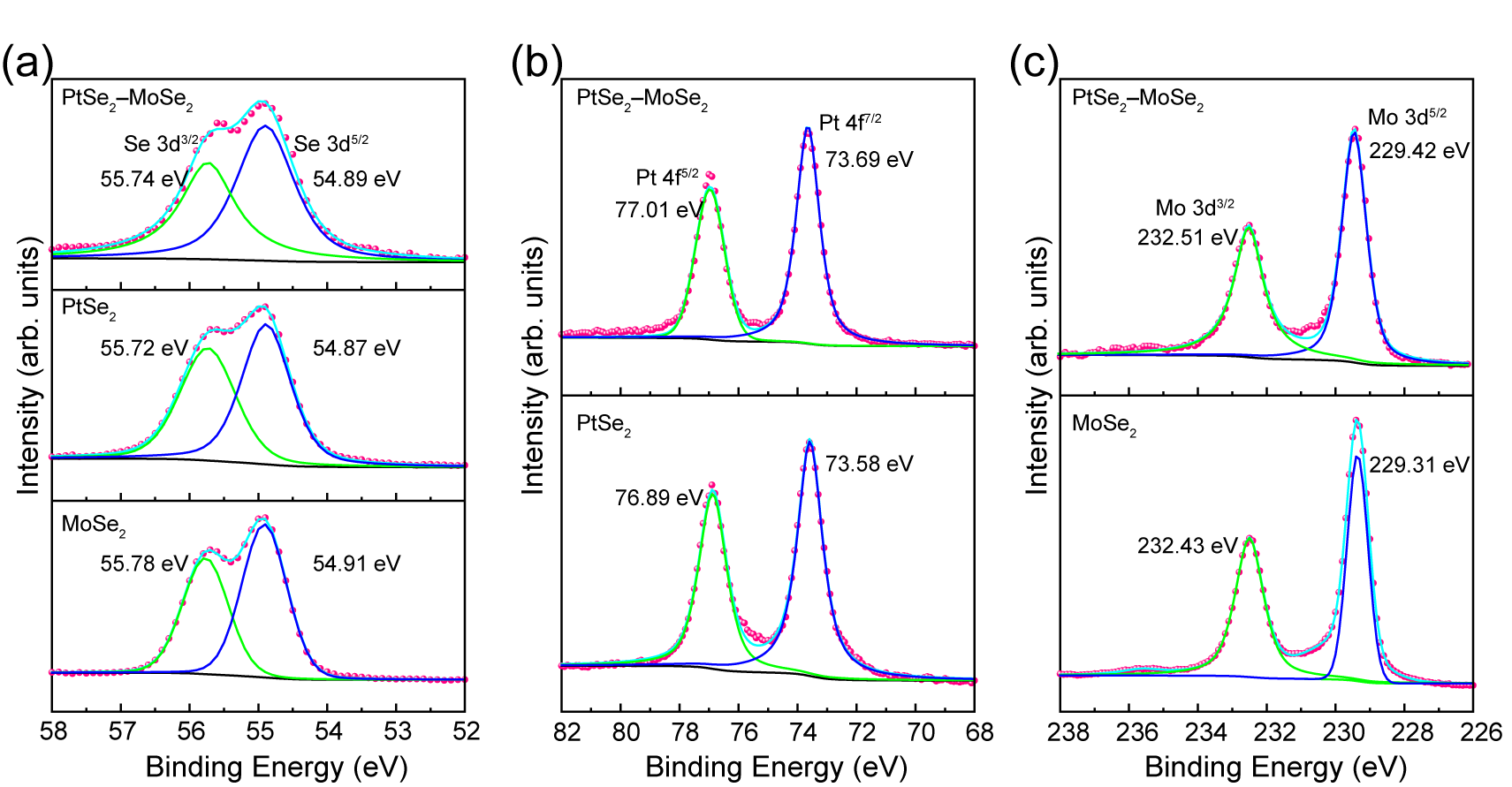


**Figure S1.** XPS characterizations of PtSe_2_**–**MoSe_2_ heterostructure, pure PtSe_2_ and pure MoSe_2_. a-c) The comparison of the core-level binding spectra of Se 3d, Pt 4f and Mo 3d obtained from the above samples, respectively.

## Section S3. The control TR experiment for sample on the SiO_2_ substrate

The noisy background for the signal from ultrafast pump-probe spectroscopy due to the metallic nature of BLG brings, inevitably decreases the S/N ratio in the time-resolved measurements, which are confirmed by the control experiment performed on a MoS_2_/SiO_2_ sample under the same experimental conditions. In this control measurement, the spectra with the intensities of more than 10^-2^ indicate high quality measurements from our home-built pump-probe spectroscopy system. Thus, we believed that the low S/N ratio can be attributed to the background signal noise from the BLG-SiC (0001) substrate.

**
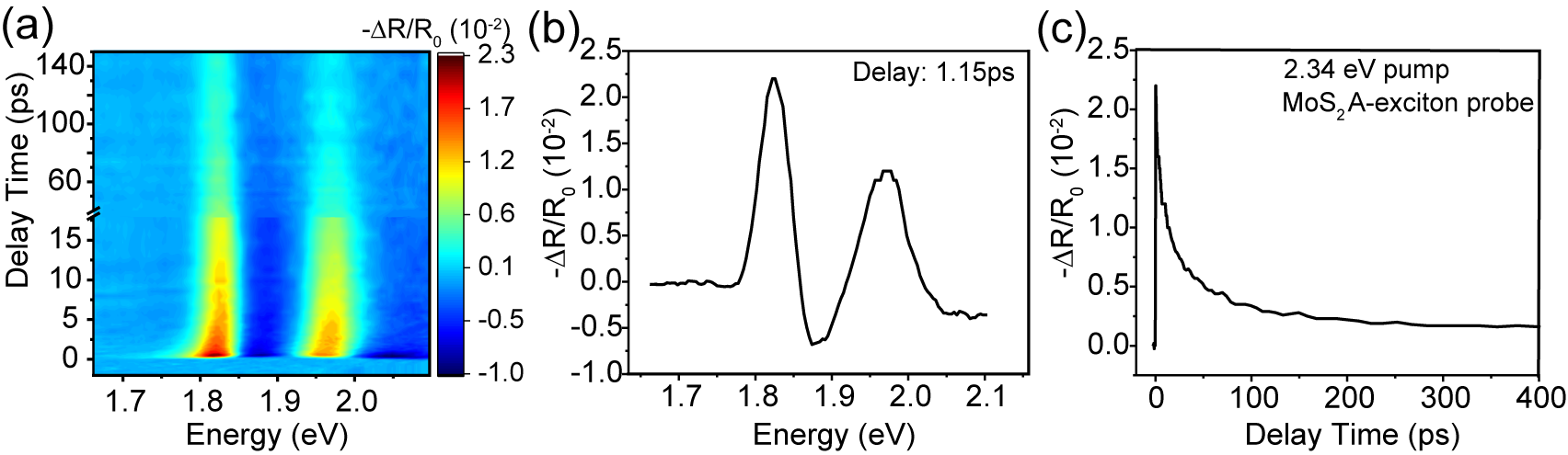
**

**Figure S2.** a) TR contour plot of a MoS_2_ on SiO_2_ with a 2.34 eV pump pulse. b) TR spectra extracted from (a) with the delay of 1.15 ps. c) Kinetics of the A-exaction resonance derived from (a).

## Section S4. Additional data for TR measurements


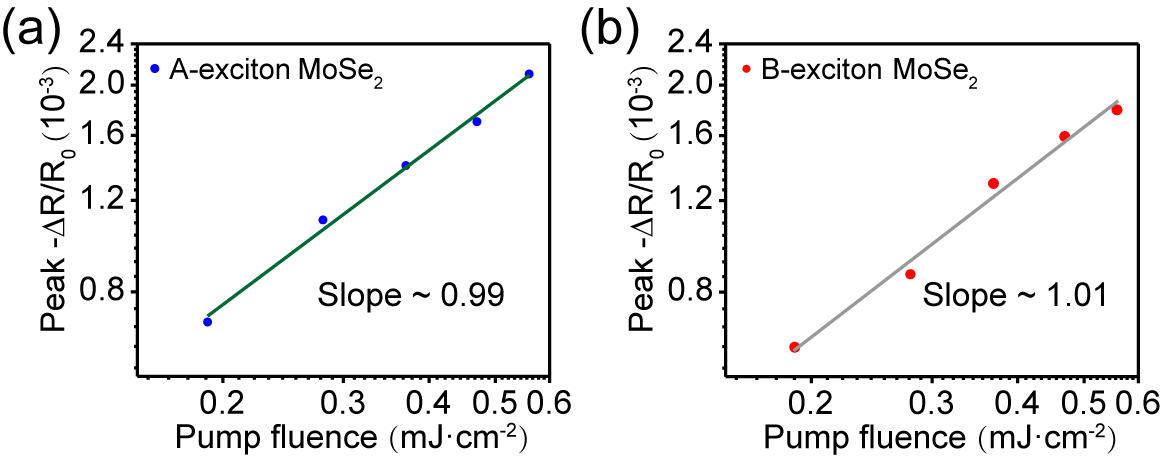


**Figure S3**. a) and b) Pump fluence dependent kinetics intensity of a PtSe_2_**–**MoSe_2_ heterostructure with the excitation at 1.26 eV, in which the amplitude of MoSe_2_ A and B-exciton resonant response scaled linearly with the pump fluence. The dark-green and gray lines indicate a linear fit, those slopes are shown as values in the Figure, respectively.

## Section S5. Topographic characterization of MoSe_2_ and PtSe_2_–MoSe_2_ heterostructure on a BLG-SiC (0001) substrate

Figures S4a and S4b show the topography of the pure MoSe_2_ and PtSe_2_–MoSe_2_ heterostructure samples used for TR measurements, respectively. The dashed line indicates the region where ML PtSe_2_ grows in-plane along the ML MoSe_2_, which approximately represent the lateral heterojunction area. The area ratio of dashed area is approximately 36 %, a calculation derived from the labeling of the grid with pixel point values of 66984 and 115359, respectively. This implies the relative proportion of the lateral to the vertical to some extent.

**
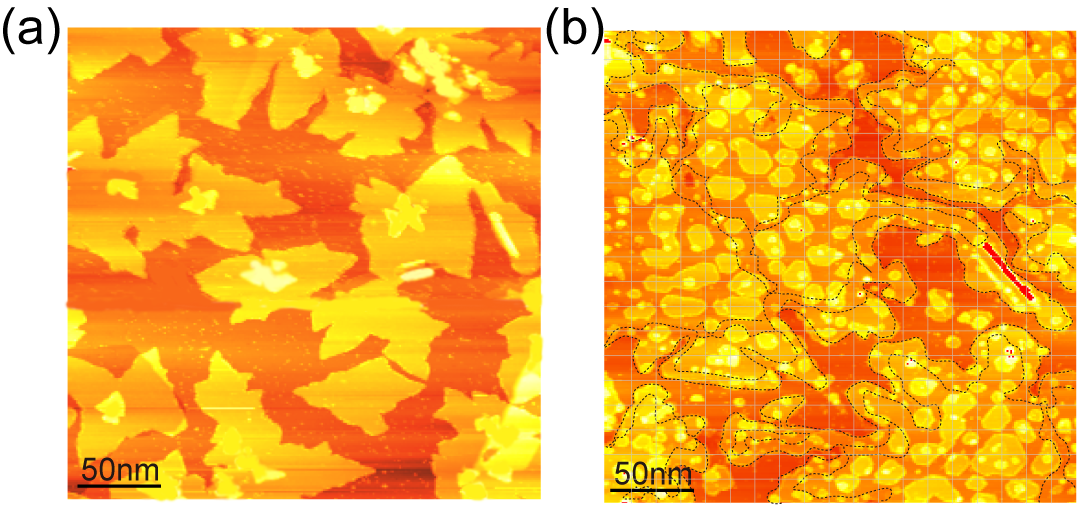
**

**Figure S4.** STM topography image for a) pure MoSe_2_ and b) PtSe_2_–MoSe_2_ heterostructures. V_b_ = 3.0 V, I_t_ = 10 pA.

## Section S6. Thickness characterization of PtSe_2_ and MoSe_2_ monolayers on a HOPG substrate

Thickness characterization of PtSe_2_ and MoSe_2_ monolayers were performed on a HOPG substrate. And the bottom panels show the height profiles along the dashed line marked in the corresponding upper panel. Both profiles demonstrate a height difference of around 1 Å, which is approximately the same value as that measured for a lateral PtSe_2_**–**MoSe_2_ heterojunction. Those results indicate that the geometric height difference does exist at the interface.


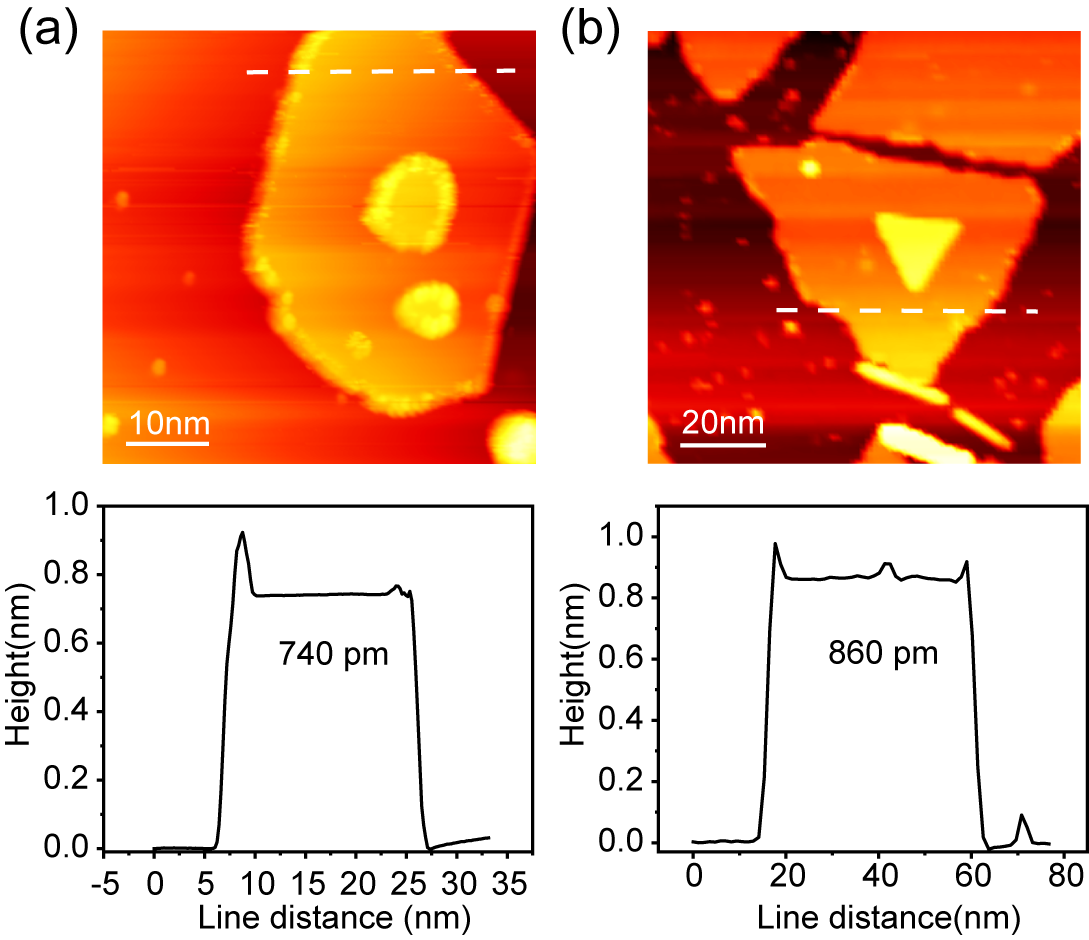


**Figure S5**. STM images of a) monolayer PtSe_2_ and b) monolayer MoSe_2_ grown on a HOPG substrate, respectively, V_b_ = 3.0 V, I_t_ = 10 pA.

## Section S7. STM and corresponding FT-STM images of the heterostructures and TMD monolayers

The FT-STM images of the monolayer and heterojunction regions were obtained, respectively, as shown in Figure S6. These measurements reveal that two sets of the reciprocal lattices in Figure S6e are constituted with the isolated MoSe_2_ (red circles) and PtSe_2_ (white circles), which confirms the fabrication of lateral heterojunction. And the lattice plane orientation of PtSe_2_ is misaligned with that of MoSe_2_. Unexpectedly, two different sets of reciprocal lattices were identified in Figure S6h, corresponding to PtSe_2_ (white solid circles) and MoSe_2_ (red solid circles) with certain angles, which suggest that rotational mismatch is present between the top PtSe_2_ layer and the bottom MoSe_2_ layer.


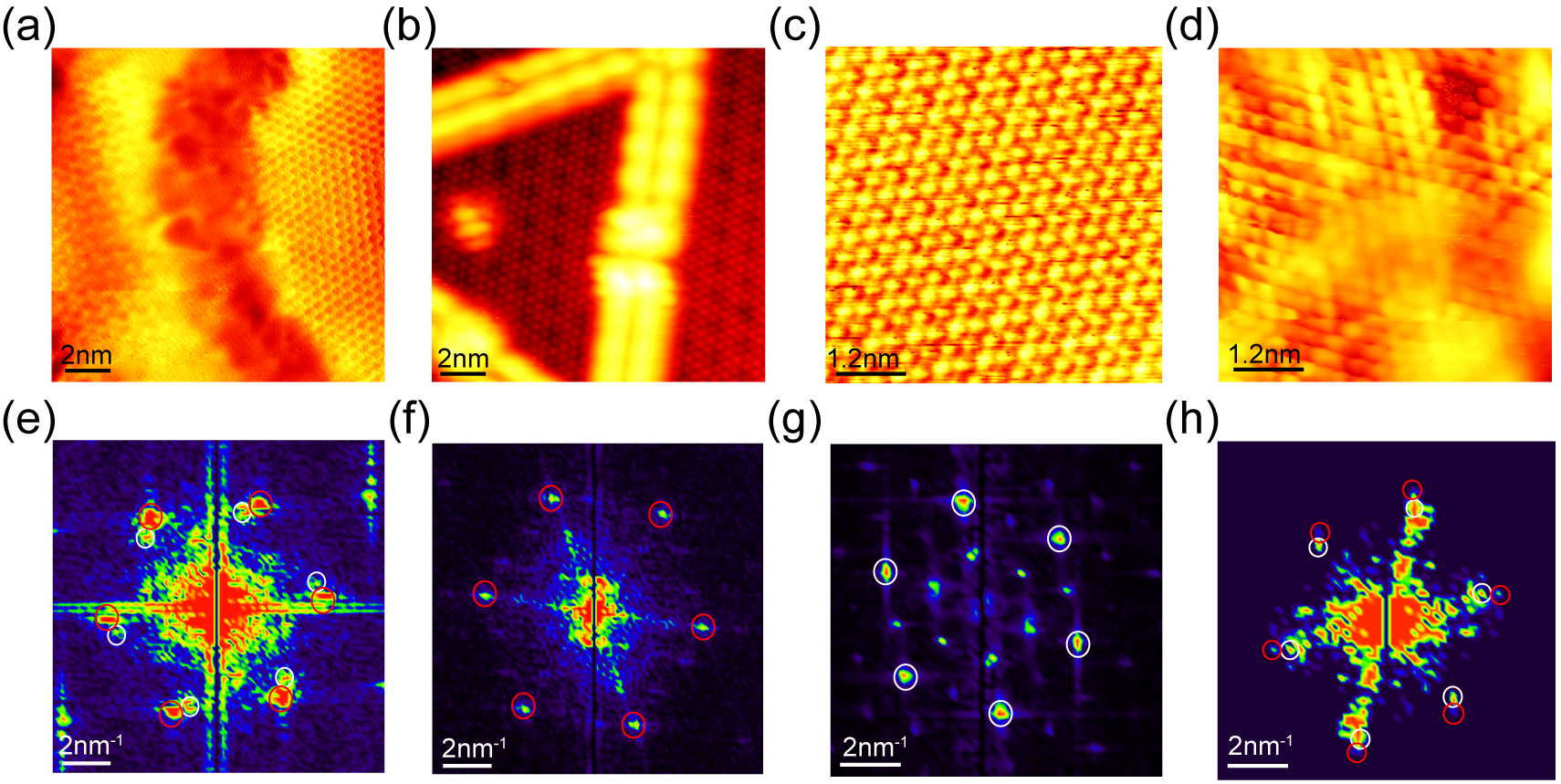


**Figure S6**. a) The dI/dV map of a lateral PtSe_2_–MoSe_2_ heterojunction at the interface, in the same area as Figure 4b in the manuscript. b) and c) are the atomic-resolved STM images of isolated monolayer MoSe_2_ and PtSe_2_. The FT-STM images. d) An atomic-resolution STM image of the PtSe_2_ surface in a vertical PtSe_2_/MoSe_2_ heterojunction, corresponding to the upper inset in Figure 4c in the main text. e-h) are transformed from the corresponding upper panel (a-d). a), **−**1 V, 180 pA; b), **−**1 V, 150 pA; c), **−**0.6 V, 200 pA; d), **−**1.1V, 80 pA.

## Section S8. STM images of the lateral heterostructure interface

Due to the different lattice symmetries and large lattice parameter difference, the interface between PtSe_2_ and MoSe_2_ is so messy. Great efforts were taken to collect STM images at different biases at this area, but no atomic structure was observed at the interface. It can be seen that the interface electronic state shows a significant dI/dV intensity change with the variation of the bias voltage. Such strong interfacial electron scattering is exceptionally challenging for the acquisition of atomic resolved images.


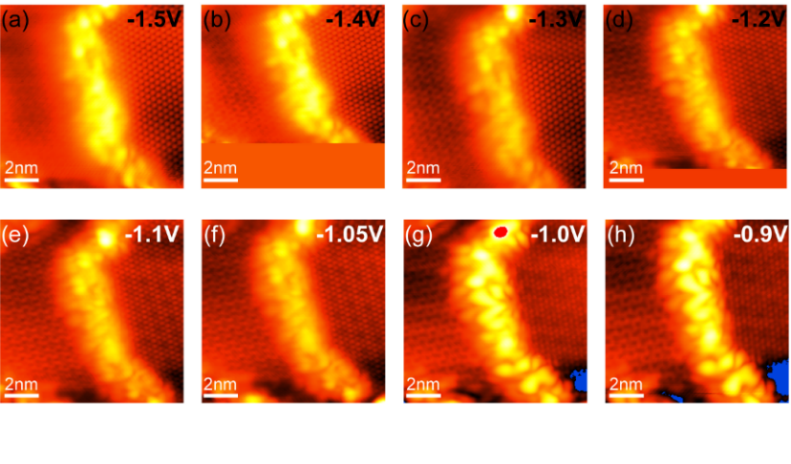


**Figure S7**. a-h) Atomically resolved STM images of the same interface region at different bias voltages.


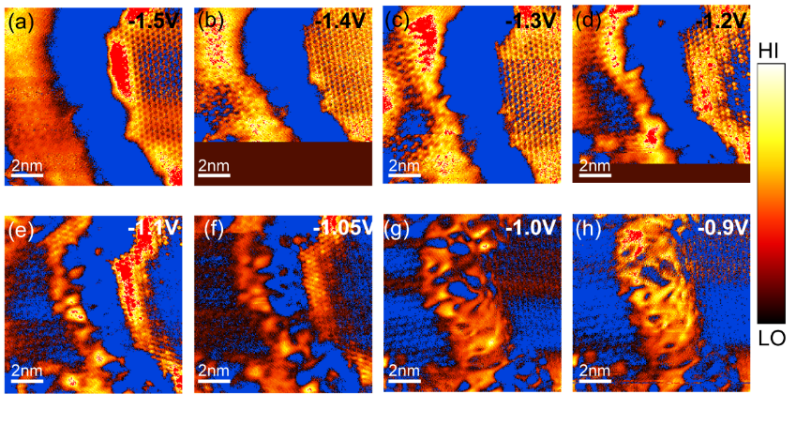


**Figure S8.** a-h) STS maps of the same interface region at different bias voltages.

## Section S9. The effect of tip induced band bending (TIBB)

Due to the poor screening of electric field between tip and sample surface, TIBB might change the energetic location of the electric band structure in STS measurements for monolayer TMDs. The typical way to rule out TIBB as a source of error in the bandgap is to vary the tip-sample distance by fixing the set-point voltage and adjusting the set-point current value ^[1]^. As shown in Figure S9a, we show several STS spectra obtained with different set-point current values (set-point voltage, V_s_ = 1 V). What’s more, we show statistical distributions of the results from 90 individual tunnelling spectra measured from different lateral position in Figure S9b, with the same set-point voltage and current value. We find that TIBB induce extremely slight shifts of the band edges for the set-point current rang less than 0.5 nA, and the observed errors are within the range of bandgap errors under lateral positional variations. Thus, we assume that the TIBB-induced errors in determining the bandgap in our experiments are approximately the same relative to the errors at different lateral position. Therefore, combining Figures S9a and S9b deduces that the mean value and the standard deviation of the quasi-particle bandgap of the monolayer MoSe_2_ is 2.19 ± 0.04 eV. Similarly, we show the statics distribution of bandgap values for monolayer PtSe_2_ (Figure S9c) and MoSe_2_/PtSe_2_ vertical heterojunctions (Figure S9d) results from the 90 individual STS spectra in different regions, yielding band gap values of 1.87 ± 0.03 eV for PtSe_2_ and 1.69 ± 0.02 eV for vertical heterojunctions, respectively. Hence, the uncertainties that arise from TIBB as well as lateral positional variations are combined in the standard deviation of our reported bandgap measurements.


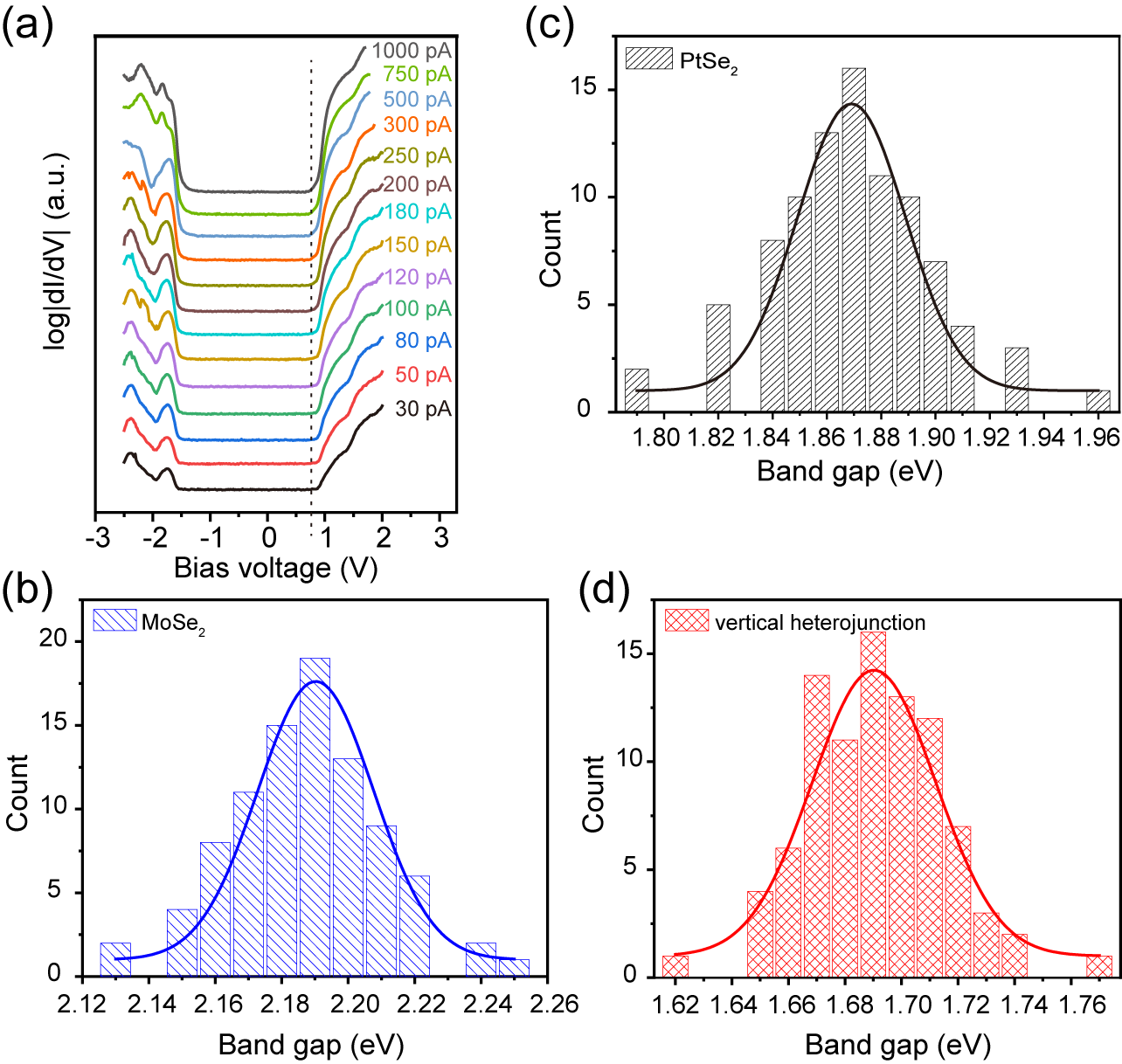


**Figure S9.** Scanning tunnelling spectroscopy of MoSe_2_, PtSe_2_, and vertical heterojunction. a) dI/dV spectra of monolayer MoSe_2_ acquired at different set-point currents (V_s_ = 1 V). b-d) Statistical distribution of band gap measured for monolayer MoSe_2_, monolayer PtSe_2_, and MoSe_2_/PtSe_2_ vertical heterojunction (from 90 individual dI/dV spectra). V_s_ = 1 V, I_t_ = 100 pA.

## Section S10. The interfacial states and band bending for the vertical heterojunction

The STS measurements show that combined effect of band bending and the interface states of vertical heterojunction, exhibiting a gap value of ~ 0.2 eV at the interface, with VBM and CBM located roughly at **−**0.039 to 0.158 eV (Figure S10a). Such semi-metallic states at the interface are suggested to be caused by metallic edge states of the top PtSe_2_ layer.^[2]^ In addition, the effect of interlayer interactions on the electronic structure cannot be ignored. For ML PtSe_2_, the VBM at the Γ point has been shown to be sensitive to the lattice strain.^[3]^ As it is known that the interlayer interaction is likely to be modified by the lattice strain, which usually has a maximum at the interface when the significant differences in the lattice constants of the two materials are present, as previously reported for WSe_2_/MoS_2_.^[4]^ From spectra #5 to #11, the locations of the VBM and CBM both are gradually shifted away opposite to the Fermi level while keeping the shape of the spectral line, where the band bending effect would be ascribed to the presence of interfacial electronic state.

Moreover, it can be observed that band bending primarily occurs in the heterojunction region. From the reported calculation results,^[5]^ the hybridized interfacial states of the heterostructures are contributed from the p_z_ orbitals of the interfacial Se atoms, in combination with Pt and Mo atoms’ d_z_^2^ orbitals. In other words, the electronic states of the bottom MoSe_2_ might be little affected by that of the outer edge of the top PtSe_2_ layer, hence the energy band bends only at the side of the heterojunction. Furthermore, the effect of domain boundary states of MoSe_2_ (STS #28 in Figure S10b) on the electronic structure of the heterojunction (STS # 1 in Figure S10b), especially the new electronic states appearing in the VB and bandgap.


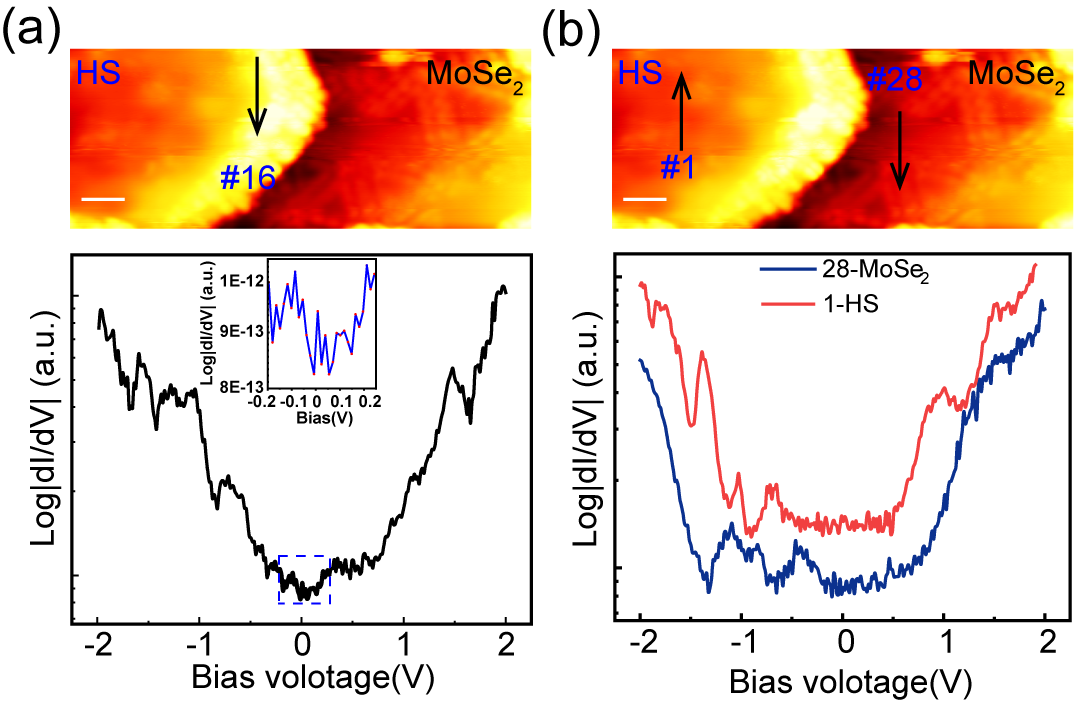


**Figure S10**. (a) The dI/dV spectrum #16 was acquired from the vertical heterojunction interface as indicated by the arrow in upper panel. (b) The dI/dV spectra #1 and #28 were collected from the top PtSe_2_ layer and the bottom MoSe_2_ layer those far away from the interface, but lied on the domain boundary, as indicated by the arrows in the upper panel.

**References**

[1] M. M. Ugeda, A. J. Bradley, S. F. Shi, F. H. da Jornada, Y. Zhang, D. Y. Qiu, W. Ruan, S. K. Mo, Z. Hussain, Z. X. Shen, F. Wang, S. G. Louie, M. F. Crommie, *Nat. Mater.* **2014**, 13, 1091.

[2] J. Li, T. Joseph, M. Ghorbani‐Asl, S. Kolekar, A. V. Krasheninnikov, M. Batzill, *Adv. Funct. Mater.* **2022**, 32, 2110428.

[3] S. Deng, L. Li, Y. Zhang, *ACS Appl. Nano Mater.* **2018**, 1, 1932.

[4] C. Zhang, M. Y. Li, J. Tersoff, Y. Han, Y. Su, L. J. Li, D. A. Muller, C. K. Shih, *Nat. Nanotechnol.* **2018**, 13, 152.

[5] a)L. Xiang, Y. Ke, Q. Zhang, *Appl. Phys. Lett.* **2019**, 115, 203501; b)J. Zhou, X. Kong, M. C. Sekhar, J. Lin, F. Le Goualher, R. Xu, X. Wang, Y. Chen, Y. Zhou, C. Zhu, W. Lu, F. Liu, B. Tang, Z. Guo, C. Zhu, Z. Cheng, T. Yu, K. Suenaga, D. Sun, W. Ji, Z. Liu, *ACS Nano* **2019**, 13, 10929.
